# Two genomes are better than one: history, genetics, and biotechnological applications of fungal heterokaryons

**DOI:** 10.1186/s40694-016-0022-x

**Published:** 2016-05-04

**Authors:** Noah B. Strom, Kathryn E. Bushley

**Affiliations:** Department of Plant Biology, University of Minnesota, 826 Biological Sciences, 1445 Gortner Avenue, Saint Paul, MN 55108 USA

**Keywords:** Heterokaryon, Parasexual cycle, Complementation, Vegetative compatibility groups, Heterosis, Protoplast fusion, Biotechnology

## Abstract

Heterokaryosis is an integral part of the parasexual cycle used by predominantly asexual fungi to introduce and maintain genetic variation in populations. Research into fungal heterokaryons began in 1912 and continues to the present day. Heterokaryosis may play a role in the ability of fungi to respond to their environment, including the adaptation of arbuscular mycorrhizal fungi to different plant hosts. The parasexual cycle has enabled advances in fungal genetics, including gene mapping and tests of complementation, dominance, and vegetative compatibility in predominantly asexual fungi. Knowledge of vegetative compatibility groups has facilitated population genetic studies and enabled the design of innovative methods of biocontrol. The vegetative incompatibility response has the potential to be used as a model system to study biological aspects of some human diseases, including neurodegenerative diseases and cancer. By combining distinct traits through the formation of artificial heterokaryons, fungal strains with superior properties for antibiotic and enzyme production, fermentation, biocontrol, and bioremediation have been produced. Future biotechnological applications may include site-specific biocontrol or bioremediation and the production of novel pharmaceuticals.

## Background

Heterokaryosis refers to the presence of two or more genetically distinct nuclei within the same cell. While uncommon throughout most of life’s kingdoms, heterokaryosis is a hallmark of kingdom Fungi. The fungal subkingdom, Dikarya, which contains two phyla (Ascomycota and Basidiomycota) and 95 % of all known fungal species [1], is named for its characteristic heterokaryons with exactly two genetically distinct nuclei. Basidiomycota are often distinguished by the presence of clamp connections, a cytological feature unique to this phylum that helps to ensure the segregation of both haploid nuclei into daughter cells following mitosis. Ascomycetes are often characterized by croziers, structures similar to clamp connections, that maintain the dikaryotic state of ascogenous cells [2]. In the sexual cycle, the nuclei in these dikaryotic cells fuse and undergo meiosis, resulting in genetically recombined haploid basidiospores or ascospores.

**Fig. 1:**
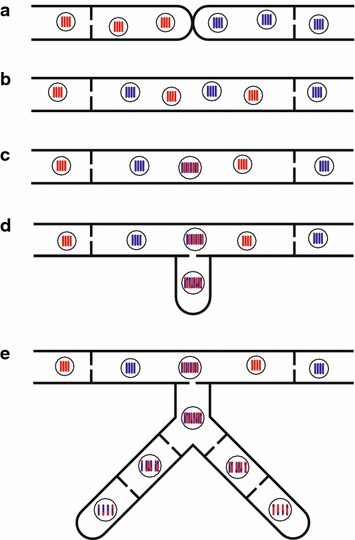
The parasexual cycle. The parasexual cycle parallels events in the sexual cycle, resulting in genetically unique haploid offspring but without a meiotic reduction. **a** Hyphae of genetically unique homokaryotic parents grow towards each other by chemotaxis and fuse. **b** Nuclei from each unique strain migrate within the fused hypha, which is now considered a heterokaryon. **c** Haploid nuclei in the heterokaryon undergo karyogamy to create a heterozygous diploid nucleus. **d** The diploid nucleus undergoes mitotic recombination to produce a recombined genotype. **e** In growing hyphae, gradual loss of chromosomes due to repeated rounds of mitotic non-disjunction results in haploidization and unique genotypes in various sectors of mycelium

Heterokaryosis also occurs during vegetative growth as a component of the parasexual cycle, a mechanism for increasing genotypic diversity in predominantly asexual fungi (Fig. 1) [3]. In this paper, the term “predominantly asexual” is used to refer to fungi that historically were not known to have a sexual cycle and those that only rarely undergo sexual recombination. In the parasexual cycle, first described in 1956 by Pontecorvo [4], hyphae from two compatible individuals grow towards each other by chemotaxis [5] and fuse, allowing exchange of nuclei to form a heterokaryon [4]. These nuclei become mixed in the cytoplasm of the heterokaryon [6] and sometimes undergo karyogamy, resulting in diploid cells. Following karyogamy, mitotic recombination and repeated chromosome loss through mitotic nondisjunction result in the formation of haploid, as well as some aneuploid, cells with unique genomes from those of either parent nucleus [4]. Thus, even in the absence of meiosis and sexual reproduction, the parasexual cycle is effective in increasing genotypic diversity in predominantly asexual fungi [7].

**Fig. 2:**
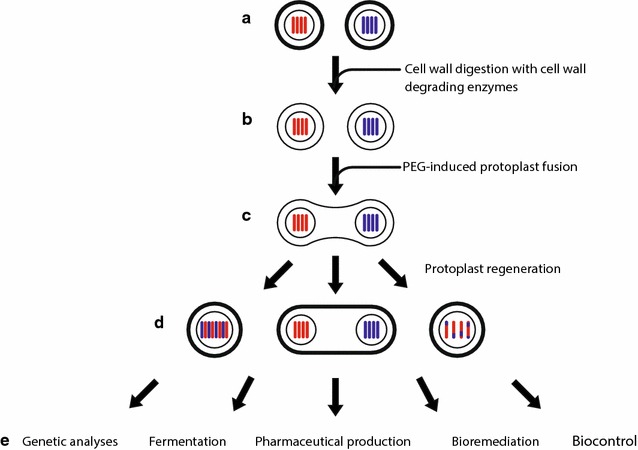
Protoplast fusion. **a** Fungal cell walls are digested with cell wall degrading enzymes, exposing protoplasts (**b**). **c** Polyethylene glycol (PEG) facilitates fusion of protoplasts, which may belong to different strains or species, as represented by red or blue chromosomes. **d** Fused protoplasts form heterokaryons and may progress partway or completely through the parasexual cycle, resulting in diploid or recombined haploid hybrids. **e** Applications of fungal heterokaryons or hybrids resulting from protoplast fusion include genetic analyses, fermentation, pharmaceutical production, bioremediation, and biocontrol

Fungal biologists have taken advantage of heterokaryosis during the parasexual cycle to enable genetic analyses and to advance biotechnology. For example, heterokaryons have enabled gene mapping, complementation, and epidemiological studies in predominantly asexual fungi. The power of heterokaryosis has also been harnessed to create beneficial fungal hybrids in applications as diverse as antibiotic production and bioremediation (Fig. 2). This review focuses on the evolution of scientific thought about non-sexual heterokaryosis, its biotechnological applications, and future directions for basic and applied research.

## Early discoveries

The term, heterokaryosis, was coined in 1912 by German mycologist, Hans Burgeff [8]. Working with *Phycomyces nitens*, he noticed that different sporangiospores could give rise to phenotypically distinct mycelia [8, 9], contrary to the phenotypically identical offspring one would predict from asexual mitotic reproduction. In 1932, Hansen and Smith, working with *Botrytis cinerea*, showed that the phenotypically distinct mycelia arose from either one or the other haploid nucleus or from both, which were variably present in the sporangiospores [10]. Their explanation, now known to be correct, was that dissimilar nuclei of the heterokaryotic mycelium segregated into different cells, resulting in homokaryotic regions of the mycelium that differed from each other in their genetic makeup.

In 1944, Beadle and Coonradt, working with *Neurospora crassa*, produced two auxotrophic strains, one of which could not synthesize p-aminobenzoic acid, and the other of which could not synthesize nicotinic acid [11]. When grown separately on minimal media, neither strain thrived, but when grown together on minimal media, hyphal fusion allowed for the formation and growth of a heterokaryon possessing a functional copy of each gene deficient in the other strain. This genetic complementation experiment suggested the potential advantages of heterokaryosis in wild asexual fungi and a rationale for the persistence of heterokaryons throughout fungal evolution.

## Heterokaryons and adaptation

Beadle and Coonradt also suggested that nuclear exchanges may take place multiple times in the lifetime of a single fungus, allowing fungi to continually update their genomes through contact with compatible neighbors [11]. This potential for genetic variation within the lifetime of a single fungus was hypothesized to play an important role in the fungus’ ability to adapt to a continuously changing environment. In 1952, Jinks provided the first experimental data to support the hypothesis that genotypically distinct nuclei within the same mycelium could be involved in adaption [12]. In a culturing experiment involving various types of media, he showed that the ratios of two genotypically unique nuclei within a wild strain of *Penicillium* fluctuated according to media type. Similar unbalanced ratios of nuclei have been observed in the basidiomycete, *Heterobasidion parviporum* [13]. Whether these shifts resulted from selection acting on nuclei [14] or from more random processes of gain or loss in particular mycelial fractions requires further investigation. Recently, it has been shown that heterokaryons and subsequent hybrids can form between different strains of the grass endophyte, *Epichloë*, through vegetative hyphal fusion [15]. In fact, the existence of multiple genotypically distinct nuclei within the same mycelium may be more common than is recognized, particularly among filamentous ascomycetes [16]. This nucleotypic diversity allows for increased phenotypic plasticity and a complex interplay of coordinated competition and/or cooperation among nuclei [14, 16, 17]. Additionally, the parasexual cycle, even when aborted by heterokaryon incompatibility reactions, provides a possible mechanism for the transfer of DNA elements, such as supernumerary chromosomes, between normally incompatible strains [18–20].

As presumed asexual organisms [21], arbuscular mycorrhizal fungi (AMF) pose a conundrum as to how they have escaped the accumulation of deleterious mutations and thrived over such long evolutionary time scales. It remains controversial as to whether AMF, ubiquitous plant root symbionts whose spores are multinucleate [22], are homokaryotic or heterokaryons formed through hyphal fusion [23, 24]. However, research suggest that selective forces acting among nuclei and the mechanisms of spore biogenesis involved in sorting of nuclei into spores may serve to buffer against mutational load [22]. This process of differential segregation of distinct nuclei into spores may also provide a mechanism for adaptation. An experiment with a strain of *Rhizophagus irregularis* showed that switching of plant hosts resulted in an adaptive shift in nucleotype frequency [25]. Nucleotypes that made the fungus better adapted to the new plant host became more common while nucleotypes with less adaptive genes decreased in frequency, allowing certain segregants to be more effective colonizers of their new hosts. A related study showed that segregation of nucleotypes in an AMF that normally had no effect on plant growth resulted in an AMF capable of inducing rice growth at five times the rate of a control [26].

Expression of genes from multiple genetically distinct nuclei within the mycelium of a single AMF results in extremely diverse transcriptional profiles. These profiles include expression of tens or even hundreds of divergent alleles [27], which likely play a role in the phenotypic plasticity of AMF. Future genomic and labeling studies of individual nuclei in AMF will hopefully clarify how genotypic diversity is partitioned among nuclei and provide insights into the mechanisms behind host recognition [28] and growth promotion. Given the fundamental role of AMF in plant root physiology, these findings will have far-reaching implications for agriculture and sustainable food production.

## Gene mapping and complementation tests

Pontecorvo was the first to recognize the potential use of the parasexual cycle for genetic mapping in fungi for which a sexual cycle has not been identified or for those with an infrequent sexual cycle [4]. In 1958, Käfer developed a method for counting the number of linkage groups and for mapping genes along the 8 chromosomes in the sexual species, *Aspergillus nidulans*, that utilized mitotic haploidization and mitotic recombination, rather than meiotic recombination [29]. In order to determine the overall number of chromosomes, he simply counted the number of groups of completely linked genetic markers among the haploid segregants of diploids carrying multiple genetic markers. Markers that always segregated together were considered to be located on the same chromosome. In order to map a gene to a specific linkage group, he formed heterokaryons between a tester strain for which specific linkage groups were marked and a mutant strain carrying the mutant gene. Diploids resulting from karyogamy were identified on the basis of spore color and auxotrophic markers. Haploid segregants of these diploids showed segregation in repulsion between the mutation and the genetic marker of one linkage group but independent segregation with markers of all other linkage groups. In this way, genes were mapped to specific chromosomes.

Pontecorvo and Käfer’s method for mapping the relative locations of genes on specific chromosomes involved several steps [30]. First, they selected genetic markers in one linkage group that showed a different phenotype in a heterozygous compared to a homozygous state. Then, heterokaryons were formed between individuals with these contrasting genetic markers, and their corresponding heterozygous diploids were isolated. If mitotic recombination occurred in these diploids, this led to some diploid segregants that were homozygous for one or more of these markers. By observing which combinations of genetic markers were homozygous in the recombined diploid segregants, the order of the marked genes along one chromosome arm could be determined. By combining this information with the results of mitotic haploidization experiments, which map genes to specific chromosomes, two chromosome arms could be assigned to the same chromosome, forming a map of an entire linkage group, including the location of the centromere.

Pontecorvo and Käfer’s methods [30] opened the door to mapping genomes of predominantly asexual species in which the parasexual cycle was thought to provide the principle means of genetic recombination. Many researchers have created genetic maps using these methods [31–33]. For example, eight linkage groups in *Aspergillus niger* were identified [33] and the location of marker genes in all eight of these linkage groups was mapped using methods similar to Käfer’s [34]. These methods have also been used to create genetic maps of linkage groups in *Penicillium chrysogenum* [35] and *Aspergillus parasiticus* [36].

In addition to genetic mapping, heterokaryons allow dominance and complementation testing in predominantly asexual fungi [11, 37]. Similar to the genetic mapping protocols, the dominance test involves pairing of a mutant strain to a wild type strain to form a heterokaryon [37]. In this test, a gain-of-function or other type of dominant mutation is indicated if the fusant expresses the mutant, rather than wild, phenotype. This protocol has been utilized in various fungal genetics studies [38, 39], including one that demonstrated incomplete dominance of an azole resistance gene [40].

The complementation test involves forming heterokaryons between two mutant strains to test if recessive mutations are in the same gene. Genetic complementation results in a normal phenotype if recessive mutations in the two strains are on different genes, whereas heterokaryons retain the mutant phenotype if the recessive mutations are in the same gene in both sets of haploid nuclei. Complementation tests are often used to visualize heterokaryon formation between two fungal isolates expressing different genetic or auxotrophic markers, as in tests of vegetative compatibility. Beginning in the 1960s, new interest arose in complementation experiments in *Phycomyces*, the same genus in which heterokaryosis was first studied by Burgeff in the early part of the twentieth century. In one study, mutant strains of *Phycomyces blakesleeanus*, each of which lacked a specific enzyme in the carotenoid biosynthesis pathway, were allowed to form heterokaryons [41]. In this experiment, one parent strain was white and only produced 1 % of the beta-carotene produced by wild type strains. The other parent strain was red, because it only produced lycopene, a precursor to beta-carotene. A heterokaryon resulting from fusion of these strains produced the yellow beta-carotene pigment, a result that helped to elucidate the beta-carotene biosynthetic pathway. A later experiment demonstrated complementation between different alleles encoding the phytoene dehydrogenase enzyme involved in beta-carotene synthesis [42], demonstrating the multimeric nature of this enzyme. Heterokaryons will undoubtedly lend themselves to many other areas of genetic research in the future.

## Vegetative compatibility

Vegetative compatibility refers to the ability of vegetative hyphae from different fungal individuals to fuse and form stable heterokaryons [43]. Several different tests have been devised for determining vegetative compatibility between different fungal strains. One of the simplest methods relies on the formation of a barrage, which may take the form of a pigmented, opaque, or clear zone between vegetatively incompatible strains [44]. Absence of a barrage between strains is indicative of vegetative compatibility, though the type of media has been shown to affect the outcome of this test in *N. crassa* [45]. Other tests for vegetative compatibility rely on complementation of pigmentation [46] or auxotrophic [11, 47] markers. In 1985, Puhalla developed a new test for vegetative compatibility involving nitrate non-utilizing (*nit*) mutants, which are isolated based on their inability to reduce chlorate, a compound toxic to fungi [48]. When grown on chlorate-containing media, spontaneous mutations result in fast-growing sectors that are assumed to be chlorate-resistant and also nitrate non-utilizing, because nitrate is assimilated by the same metabolic pathway as chlorate [49, 50]. One benefit of this system is that mutagens need not be used to generate auxotrophic mutants. Puhalla attributed the nitrate non-utilizing phenotype of *nit* mutants in *Fusarium oxysporum* to one of two genetic mutations, which he referred to as *nitA* and *nitB* [48]. When grown on minimal media with nitrate as a sole nitrogen source, neither type of mutant thrived unless they underwent vegetative fusion to form a heterokaryon, which restored a complete nitrate reduction pathway through complementation.

Later researchers refined Puhalla’s techniques and identified four main *nit* phenotypes, which are referred to as *nit*1, *nit*2, *nit*3, and *nit*M [51, 52]. The most commonly reported phenotypes recovered are *nit*1 and *nit*M [53–55], and pairing of these is used to visualize complementation and heterokaryon formation for a variety of applications. Due to its simplicity, the use of the *nit* mutant system has become a standard test for grouping fungal isolates into vegetative compatibility groups (VCGs), which consist of all strains of a fungal species that are capable of undergoing vegetative fusion and stable heterokaryon formation with each other [46]. The *nit* mutant system also has several biotechnological applications, including the creation of hybrid biological control agents [56, 57].

Recently, a high-throughput method for obtaining *nit* mutants and performing complementation tests has been developed and tested [55]. This method speeds the generation of *nit* mutants by exposing them to ultraviolet radiation on chlorate-containing media. The complementation tests are performed in liquid media in 96-well plates, a method that saves materials, labor, and time, compared to conventional pairings on solid media. Another newly developed technique for determining VCGs dispenses with the *nit* mutant system altogether, instead relying on spectroscopic analysis to group isolates into VCGs [58]. Though not yet widely used, these new techniques may transform the way vegetative compatibility testing is performed.

We are beginning to understand how hyphae fuse. Studies of hyphal fusion in *N. crassa* have primarily involved conidial anastomosis tubes (CATs), structures that arise from germinating spores or germ tubes and fuse with each other. Several genes and proteins involved in CAT induction, chemotropism, and fusion have been identified [3, 59]. A particularly interesting aspect of CAT chemotropism is the oscillatory recruitment of proteins to the tips of plasma membranes of homing tubes. In homing CATs, the MAP kinase, MAK-2, localizes to the growing tip of one CAT, while the protein, SO, is simultaneously recruited to the growing tip of its partner [60]. The recruitment of these proteins alternates every 3–6 min in an anti-phase oscillatory pattern, which repeats up to six times before contact. Recently, a transcription factor, PP1, which is a predicted target of the MAK-2 pathway, was shown to be necessary for chemotropic interactions [61]. This transcription factor has been shown to promote expression of 16 genes involved in germling fusion, including *ham*-*11*, which is thought to act as a switch, initiating localization of MAK-2 to the plasma membrane when CAT pairs are less than 15 μm apart. As such, MAK-2, PP1, and HAM-11 have been hypothesized to be components of a positive feedback mechanism involved in chemotropic interactions. For reviews of proteins involved in hyphal fusion, see [3, 59, 62].

## Barriers to vegetative compatibility

While vegetative self-fusion is common in filamentous fungi, a significant limitation to harnessing heterokaryons for biotechnology is the failure of hyphae from different strains to fuse [63]. In 1975, Anné and Peberdy surmounted this barrier by dissolving fungal cell walls with cell wall degrading enzymes and subsequently fusing naked protoplasts together in a solution of polyethlyene glycol (PEG) (Fig. 2). Their seminal experiment showed that it was possible to fuse protoplasts of the same strain [64], while a subsequent experiment showed that it was possible to form intraspecific heterkaryons using this approach [65]. Later researchers expanded on Anné and Peberdy’s methods to form heterokaryons from protoplasts of different genera [66], opening up new avenues of research.

**Fig. 3:**
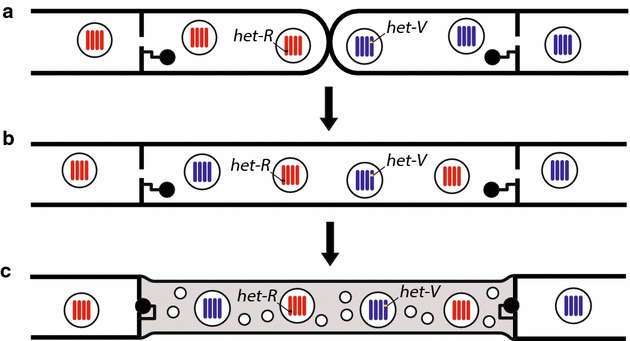
Vegetative incompatibility. **a** Hyphae of vegetatively incompatible filamentous fungi grow towards each other by chemotaxis. **b** Hyphal fusion occurs, resulting in a heterokaryon. **c** Expression of incompatible *het* genes in the heterokaryon leads to sealing of septal pores, autophagy, and programmed cell death. Vacuolization and the formation of additional septa (not pictured) also occur. Autophagy (represented by small circles in the cytoplasm of the heterokaryon) may prevent the spread of pro-death signals and is not thought to contribute to the cell death process. Thinning of the heterokaryotic filament accompanies cell death

However, even when hyphal fusion barriers are surmounted, genetic barriers to stable heterokaryon formation exist. Stable heterokaryon formation does not commonly occur between members of different fungal species or even between different VCGs of the same species in the wild, due to vegetative incompatibility (VI) [44]. VI inhibits the formation of stable heterokaryons through expression of heterokaryon incompatibility (*het*) genes, formerly referred to as vegetative incompatibility (*vic*) genes, in heterokaryotic nuclei [43, 44, 67]. When two incompatible nuclei occupy the same fungal cell, expression of their *het* genes leads to compartmentalization and cell death of the heterokaryotic cell and sometimes of neighboring cells (Fig. 3) [68].

Mechanisms of incompatibility are versatile, including both allelic and non-allelic interactions [67]. In allelic systems, genetic differences in alleles occupying the same locus result in VI. In non-allelic systems, the presence of incompatible genes at different loci leads to VI. Several VI systems have been characterized in *Podospora anserina*, *N. crassa*, and *Cryphonectria parasitica* (syn. *Endothia parasitica*). Systems characterized for *N. crassa* include the allelic mating-type associated incompatibility system and the non-allelic *het*-*c*/*pin*-*c* and *het*-*6*/*un*-*24* systems. In *P. anserina* both the allelic *het*-*s* system and the non-allelic *het*-*c*/*het*-*d* and *het*-*c*/*het*-*e* systems have been characterized [44]. The *C. parasitica* genome contains six vegetative incompatibility loci, termed *vic1*, *vic2*, *vic3*, *vic4*, *vic6*, and *vic7*, each of which contributes to an allelic VI system [69]. Recent work has focused on identifying the genes and proteins associated with these loci [70, 71]. For reviews of incompatible interactions between *het* loci, see [68, 72, 73].

VI may have evolved as a mechanism to protect filamentous fungi from the spread of parasitic nuclear genes or viruses [74]. According to this hypothesis, cell death and plugging of septal pores acts to quarantine and prevent the spread of these infectious elements. A more recent hypothesis suggests that VI may be a by-product of pathogen-driven evolution of the fungal innate immune system [75–77]. In this hypothesis, fungal pathogens, like mycophagic bacteria and mycoparasites, drive the evolution of *het* genes in much the same way that plant pathogens drive the evolution of resistance genes in plants. In support of this hypothesis is the fact that the architecture of some *het* gene products resembles the pattern recognition receptors of the plant innate immune system [76]. Furthermore, a genealogical study of *Cryphonectria hypovirus* 1 indicated that VI failed to prevent the spread of this mycovirus in *C. parasitica* [78], casting some doubt on the hypothesis that VI evolved solely to prevent the spread of viruses.

## Applications of vegetative incompatibility and compatibility

Regardless of its biological function, VI has many applications. One application of vegetative incompatibility is the identification of VCGs in plant pathogenic fungi. VCGs were originally envisioned as a proxy for races of plant pathogenic fungi, because of a rough correlation between pathogenicity or virulence and VCG [48, 54]. In plant disease diagnosis, pairing an unknown plant pathogen with a tester strain of a known VCG is a much quicker and simpler way to determine the identity of the infecting fungus compared to traditional methods of inoculating plants and fulfilling Koch’s postulates [43]. The plethora of studies on VCGs from 1983 to the present date evinces the importance of VCGs in plant pathology. However, recent studies have cast some doubt on the utility of VCGs in disease diagnosis. Individual races of *F. oxysporum* f. sp. *dianthi*, the cause of vascular wilt of carnations, and *F. oxysporum* f. sp. *cubense*, the cause of Fusarium wilt of bananas, have been shown to consist of several VCGs, each with a unique genetic profile and differing degrees of virulence [79, 80]. Furthermore, some VCGs contain not one, but several races of plant pathogenic fungi [79–81], complicating plant disease diagnosis based on VCGs. Another potential pitfall of VCG analysis is the ability of members of different VCGs to form “weak” heterokaryons [81]. While often unstable and short-lived [55], these heterokaryons have shown that exchange of genetic material is possible between members of different VCGs.

In spite of these shortcomings, VCGs have proven useful in population genetic studies. For example, by studying isolates of aflatoxin-producing *Aspergillus flavus* collected from different regions of Nigeria, researchers were able to delineate the geographic boundaries of 25 VCGs, enabling selection of appropriate non-toxic isolates of *A. flavus* to use as competitive biocontrol agents in specific regions. If biocontrol agents are in the same VCG as pathogenic fungi, it might be possible for these strains to inadvertently create heterokaryons or hybrids with increased virulence and pathogenicity [82]. Therefore, when deploying competitive biocontrol agents, it is essential to select isolates that are in different VCGs than their toxic counterparts. This study resulted in the formulation of a biocontrol agent, Aflasafe™, which has been deployed to protect maize in Africa from *A. flavus* [83].

Another remarkable application of vegetative compatibility was the development of a method for biocontrol of the chestnut blight fungus, *C. parasitica*. In 1980, Jaynes and Elliston found that by inoculating an infected tree with a mixture of hypovirulent strains of *C. parasitica* belonging to several different VCGs, the infectious mycelium was weakened [84]. This effect was less pronounced with the application of individual hypovirulent strains. It was hypothesized that a mixture of hypovirulent strains provided better control of the disease because at least some of these strains were vegetatively compatible with the infecting strain and thus able to transfer hypovirulence determinants to it. In a follow-up study, it was reported that 76 *C. parasitica* isolates from one Connecticut state forest plot fell into 35 VCGs and that transfer of hypovirulence determinants between strains was restricted to varying degrees by vegetative incompatibility barriers [85]. This research revealed an important consideration for the control of plant pathogens. If multiple VCGs of a single plant pathogen are present, biological control agents that fall into several VCGs corresponding to those of the pathogen may be required.

VI systems in fungi may even have relevance for biomedical research. While the majority of VI systems do not rely on the formation of prions, one particular system, the *het*-*s* system, involves the use of prions to alter cellular biochemistry, leading to cell death. In this system, vegetatively incompatible cells express either HET-S or HET-s proteins [75]. Upon hyphal fusion, the prion form of HET-s from the cytoplasm of one cell induces a conformational change in HET-S from the other cell, rendering this protein toxic to the heterokaryon [86]. The toxicity arises from the disruption of membrane integrity by the activated form of HET-S [87], which leads to death of the fused cells. The HET-s prion takes the form of an amyloid aggregate similar to those found in over 30 named human diseases, including Alzheimer’s, Parkinson’s, and Huntington’s disease [88, 89]. In *P. anserina*, the model organism in which VI has been most thoroughly studied, autophagy, a process whereby cellular components are sequestered and digested [90], occurs alongside cell death. Autophagy, in this case, is not necessary for the cell death process but instead may have a protective effect, scavenging pro-death signals and preventing them from triggering death of other parts of the mycelium [68]. Autophagy is also implicated in both tumor suppression and resistance to chemotherapeutics [91]. Thus, filamentous fungi, like *P. anserina*, could prove useful as model organisms for research in cancer and other human diseases.

## Pharmaceutical production

The uses of heterokaryons extend well beyond basic science, with applications in the pharmaceutical, agricultural, and environmental remediation industries. The utility of heterokaryons for a wide range of biotechnological applications likely stems from heterosis. Fungal heterokaryons often exhibit faster growth rates than either of the two parent strains [11, 92] and frequently express desirable qualities of both [56, 93]. Several genetic mechanisms may account for the improved qualities of heterokaryons [43]. One possibility is that deleterious alleles in one genome are masked by dominant or complementary alleles in the second genome [94]. Another possibility is that new gene interactions arise in the heterokaryon due to distinct allele combinations [95, 96]. Finally, hybrids between genotypically divergent parents may express novel combinations of genes or a greater diversity of transcripts, resulting in an increased variety of proteins that may confer a faster growth rate or increased production of secondary metabolites [97, 98]. The latter has been exploited for biotechnological uses. For example, in an engineered heterokaryotic strain of *Aspergillus*, each nucleotype encodes a different subunit of an antibody, which is only fully expressed in the heterokaryon [99, 100]. All of these mechanisms likely play a role in enhancing the phenotypic qualities desired by fungal biotechnologists.

In predominantly asexual fungi, the parasexual cycle has enabled the creation of hybrids with better characteristics for antibiotic production than their parent strains. An early example of this is a recombined haploid hybrid resulting from the protoplast fusion of two strains of *Acremonium chrysogenum* (syn. *Cephalosporium acremonium*) that had 40 % greater cephalosporin C production than the higher producing parent strain [101]. A second example is a hybrid formed between two *Penicillium* strains, one of which had high penicillin production but poor growth and sporulation and the other of which had low penicillin production but better growth qualities [102]. One haploid recombinant resulting from protoplast fusion of these strains produced approximately the same amount of penicillin as the high penicillin-producing parent and also expressed good growth characteristics and better sporulation than either parent. A third example is a recombined haploid hybrid created through protoplast fusion in *P. chrysogenum*, a producer of β-lactam antibiotics, that had three-fold greater penicillin production than the parent strains [103]. In this last example, rather than using traditional mutagenesis techniques to create auxotrophs, acetate non-utilizing and nitrate non-utilizing mutants were generated on fluoroacetate and chlorate media, respectively.

In spite of these successes, the literature on the use of heterokaryons for antibiotic production is scarce after 1993, possibly due to the lack of success in creating stable high-producing strains [104]. Fungi are notorious for having unstable genomes [105], and even fungi undergoing purely asexual reproduction can produce offspring with different sized chromosomes over a few generations [106]. Genetic instability of intraspecific [107] and interspecific [98] hybrids formed through protoplast fusion is a commonly reported problem. Industrial strains of *P. chrysogenum*, many of which synthesize higher levels of penicillin due to anueploidy or polyploidy, often revert to wild-type antibiotic production levels due to chromosome loss over repeated rounds of subculturing [104]. From the standpoint of fungal genetics, this instability is a natural progression of the parasexual cycle in which diploids formed from heterokaryons undergo haploidization through gradual chromosome loss (Fig. 1). Although there are methods for maintaining heterokaryons or heterozygous diploids, such as culturing prototrophic colonies on minimal media, researchers interested in strain improvement may prefer to select from the haploid segregants of heterozygous diploid hybrids rather than attempting to stabilize these diploid hybrids over time. Haploid segregants have, in many instances, yielded stable and useful recombinants [101–103, 108].

Antibiotic resistance is one of the most pressing concerns faced by microbiologists today [109], presaging the need for new antibiotics. While fungal protoplast fusion experiments have, so far, failed to generate novel antibiotics, two results of these experiments point to the likelihood of discovering novel antibiotics in heterokaryons, stable heterozygous diploids, or their recombined haploid segregants. First, fusion of non-antibiotic producing strains has resulted in antibiotic producing hybrids [98, 102]. This was shown in an experiment involving fusion of a penicillin-blocked mutant of *P. chrysogenum* with an extremely low penicillin-producing strain of *Penicillium patulum*. Fusion of these strains resulted in hybrids capable of producing penicillin at a rate up to 30-fold greater than either parent [98]. In another experiment, two penicillin non-producing auxotrophic mutants of *Penicillium* were fused, resulting in hybrids that produced penicillin, albeit at a lower rate than the grandparent strain [102]. Thus, even for strains that have no history of antibiotic production in isolation, their fused heterokaryons may, through complementation of biosynthetic pathways, produce antibiotics. Secondly, in many fungal hybrids, expression of both sets of biosynthetic genes leads to the production of secondary metabolites from both strains [41, 98]. Given that only a small fraction of the estimated 1.5 million species of fungi have been described [110] and that only a handful of these have been subjected to protoplast fusion experiments, it seems probable that by combining genomes of these fungi, new biosynthetic pathways, and therefore, new antibiotics, could be synthesized. Furthermore, novel antibiotics have been produced by protoplast fusion of bacteria [111, 112], suggesting this approach may eventually prove fruitful in fungi.

Hybridization also has the potential to improve strain production of other pharmaceuticals. Ergot alkaloids, which are used to control bleeding during labor [113] and in the treatment of migraine headaches [114], are produced by fungi in the genus *Claviceps*. While not currently being researched using hybridization techniques, two examples illustrate how protoplast fusion could be used to improve strains of this pharmaceutical-producing fungus. In one study, protoplast fusion was used to make an intraspecific hybrid of an ergotamine-producing and a clavine-producing strain of *Claviceps purpurea* [115], resulting in a hybrid that produced both alkaloids. In another study, researchers used protoplast fusion to create intraspecific hybrids that produced 30 % more alkaloid than parent strains [116]. Interestingly, the hybrids produced from the protoplast fusion of strains not carrying genetic markers produced ten times more alkaloid than hybrids formed between genetically marked strains, suggesting that traditional mutagenesis may not be the best approach when designing hybridization experiments.

Taxol, which was originally isolated from the bark of an endangered tree, *Taxus brevifolia* [117], is a drug used in the treatment of breast, uterine, and other cancers. In the quest for a cheaper and more readily available source of this drug, researchers discovered taxol-producing endophytic fungi, which were engineered to produce more taxol through mutagenesis [118]. High taxol-producing mutant strains of *Nodulisporium sylviforme* have been hybridized via protoplast fusion, resulting in improved strains with 19.35–24.51 % greater taxol production than parent strains [119] or subjected to multiple rounds of protoplast fusion and genome shuffling to create improved strains with 31.52–44.72 % higher taxol production than parent strains [120]. Future research in this field has great potential and may result in the creation of fungal hybrids capable of more efficient or even novel [107] drug production.

## Fermentation and enzyme production

Protoplast fusion has been used in an attempt to improve strains for a variety of fermentation applications. One such application is soy sauce production. Protoplast fusion was used to hybridize two strains of *Aspergillus sojae*, one of which was a protease hyper-producer, and the other of which was a glutaminase hyper-producer [121]. This experiment resulted in stable heterozygous diploids, some of which were good producers of both enzymes. However, subsequent interspecific protoplast fusion experiments with *A. sojae* and *Aspergillus oryzae* failed to create hybrids expressing both parental enzymes when protoplast fusion was followed by haploidization [122, 123], possibly suggesting a low rate of recombination in the heterozygous diploids. A later experiment, which used electrofusion to fuse protoplasts of *A. oryzae* and *A. niger*, resulted in hybrids with evidence of genetic recombination but unimproved protease or amylase production [124].

Protoplast fusion has been used to improve strains for ethanol fuel production. In one study, two strains of *Saccharomyces cerevisiae*, one of which had good thermotolerance and the other of which was a high ethanol-producer, were fused. The resulting heterozygous diploid hybrid had good thermotolerance and fermented molasses into ethanol at a higher rate than the high ethanol-producing parent [125]. This same hybrid strain was used in a later study to make biofuel from sweet sorghum [126]. In another study, intergeneric hybrids were produced by fusing mutant strains of *Penicillium echinulatum* and *Trichoderma harzianum*, both of which were selected for their increased cellulase production [108]. Some recombinant hybrids formed from the fusion of these strains produced more β-glucosidase, an enzyme used in the production of ethanol fuel, than either parent strain.

The many successes in using protoplast fusion to generate both intraspecific and interspecific fungal strains for enzyme production are too numerous to list, but a few key examples illustrate the utility of this approach. In one study, a recombined haploid hybrid was formed from the protoplast fusion of *Penicillium expansum* and *Penicillium griseoroseum* that produced more pectinases than either parent strain [127]. In another study, a recombined haploid hybrid with similarly enhanced pectinase production was created by fusing *Aspergillus flavipes* and *Aspergillus niveus* [128]. An interspecific hybrid formed between *Trichoderma* spp. selected for their ability to produce large quantities of chitinase or β-glucanase, enzymes important in degrading fungal cell walls, has also been created by protoplast fusion [129]. Some resulting hybrids in this study had two to three-fold increases in their production of both enzymes, which resulted in greater antagonistic ability against some plant-pathogenic fungi.

## Biocontrol of insects and nematodes

Of the many challenges faced by agricultural scientists, few are greater than that of developing effective biological control agents. Heterokaryons may offer solutions to controlling plant pathogens and pests. *Beauveria bassiana* is an important fungus in the biocontrol of insect pests, such as *Ostrinia nubilalis* (European corn borer) and *Leptinotarsa decemlineata* (Colorado potato beetle). In the last three decades, research has resulted in the creation of *Beauveria* hybrids that have the potential to be more effective biocontrol agents than wild type strains. In one study, recombined haploids created by protoplast fusion of diauxotrophic mutant strains of *Beauveria sulfurescens* and *B. bassiana* exhibited greater virulence against their insect hosts, providing evidence for the potential uses of fungal hybrids in pest control [93]. In another study, a hybrid strain of *B. bassiana* with enhanced thermotolerance was created through co-culturing and fusion of two *B. bassiana* isolates [130]. Thermotolerance is a prerequisite for commercialization of biocontrol agents.

Another desireable feature in commercial biocontrol agents is the ability to survive in dry environments. In an attempt to create strains that were both highly virulent and able to withstand extremes of humidity, researchers fused strains of the entomopathogenic fungi, *Lecanicillium muscarium* and *Lecanicillium longisporum* (syn. *Verticillium lecanii*), which are found in the commercially available biocontrol products Mycotal^®^ and Vertalec^®^, respectively [56]. Individual parent strains were either virulent at high humidity or could survive low humidity, but neither possessed both characteristics, limiting their usefulness as biocontrol agents. A later study tested hybrids formed from protoplast fusion between these strains for their ability to control several pests, including whiteflies, aphids, and soybean cyst nematodes [57]. Several hybrid strains outperformed Vertalec^®^ and Mycotal^®^ in terms of insect mortality and infection, and one hybrid strain reduced soybean cyst nematode cyst and egg density at a significantly higher rate than the commercial formulations. Our ability to utilize the parasexual cycle to create fungal hybrids that are superior biocontrol agents is still in its early stages but appears to be a promising area of research.

## Bioremediation

Fungi are uniquely well suited to cleansing soil of contaminants because their hyphae grow into the spaces between soil particles, forming expansive underground mycelial networks [131, 132]. While some naturally occurring fungi can break down environmental contaminants [132], hybrids resulting from artificial heterokaryons have proven to be more effective agents of bioremediation than their parent strains. For example, in one study, co-culturing of related strains of *Fusarium solani* isolated from DDT-contaminated soils resulted in the formation of recombined haploids that could break down DDT at a rate up to three-fold greater than parent strains [133]. Another example is an intergeneric hybrid that has been used to clean up shellfish waste [134]. This hybrid, which resulted from the protoplast fusion of high chitinase-producing strains of *T. harzianum* and *A. oryzae*, was more effective than either parent strain in degrading crustacean shells, a major food waste in India. In a world of oil spills and industrial waste, the engineering of powerful hybrid bioremediation agents may prove to be an important area of future research.

## Conclusions and future directions

Genome recombination through parasexual hybridization has the potential to enhance polygenic traits in hybrids that would be difficult to create using more targeted methods of genetic modification [124]. An additional advantage of recombining entire genomes is that a huge variety of potentially useful segregants can result from a single experiment, as opposed to generating a single genetically modified strain with traditional genetic engineering approaches. A third advantage may be that since hybridization only involves naturally occurring organisms, biotechnological products that result may gain greater acceptance by the public than genetically modified organisms.

A better understanding of heterokaryon formation and incompatibility may have implications for human health. Heterokaryons formed from fusion between tumor cells and healthy cells have been hypothesized to a play a role in metastasis [135]. Furthermore, elements of the parasexual cycle, like karyogamy and gradual chromosome loss, seem to be mirrored in tumorigenesis [136]. Neurodegenerative diseases, like Alzheimer’s disease, share features in common with the *het*-*s* vegetative incompatibility system. However, many aspects of heterokaryosis are still a mystery. While many genes and proteins involved in fusion competence, cell–cell communication, and directed growth have been described [60, 61], little is currently known about the process of plasma membrane merger [3], a necessary step preceding heterokaryosis in natural fungal populations. Future research may discover genes and proteins involved in this process, help to elucidate its mechanism at the cellular level, and lead to more efficient methods for creating fungal hybrids. Overcoming the limitations of vegetative incompatibility imposed by *het* genes may also open up further avenues of research. Could silencing of these genes allow for the efficient formation of new fungal hybrids? The applications of hybridization are numerous if incompatibility barriers can be overcome.

Future attempts at biocontrol may benefit from imitating the methods developed for chestnut blight. Mycoviruses similar to those passed between VCGs to control *C. parasitica* have recently been characterized in many plant pathogenic fungi [137]. If it were possible to incorporate a transmittable mycovirus into the genome of a pathogenic fungus, this may prove to be an effective agent of biocontrol. An additional advantage to this approach is that it may be possible to customize biocontrol for individual farmers by choosing biocontrol agents in the same VCGs as those encountered in their fields. This approach would increase the chances of transmitting the virus to pathogenic VCGs, while avoiding transmission to non-pathogenic VCGs and alleviating some environmental concerns over the use of biocontrol agents.

A similar approach might be used to increase the activity of wild fungal strains involved in bioremediation. If an engineered strain with superior bioremediation abilities were introduced into a compatible wild population, the transformed genes could, hypothetically, become more common in the wild population and increase its effectiveness in bioremediation. To design such strains, foreknowledge of fungal VCGs present in areas affected by environmental contamination would be necessary. Although there are certain ecological risks associated with introducing foreign genes into wild populations [138], such an approach may be desirable in the case of severe environmental disasters.

In the future, researchers may want to combine traditional techniques for genetic transformation with protoplast fusion to create superior heterokaryons for industrial uses [99]. In this scenario, specific desired genes would first be transformed into fungal protoplasts [139]. The transformants would then be hybridized to related strains to take advantage of heterosis. The resulting hybrids may express the desired genes and exhibit faster growth rates and other traits desirable for industrial applications.

Heterokaryosis has fascinated fungal biologists since 1912, and it continues to offer insights into the way fungi evolve and adapt. Research into this phenomenon over the last century has opened doors to our understanding of fungal genetics and has shed light into the ways fungi exploit their environment. In more recent decades, heterokaryons have been created artificially in the lab to produce hybrid fungi with superior properties in antibiotic production, biocontrol, and bioremediation. Future research is likely to find more uses of heterokaryons in biotechnology and to expand their uses to other medical and agricultural applications [140]. Heterokaryons may hold the answer to solving some of the world’s most pressing health and environmental issues and promise to intrigue mycologists for many years to come.

## Abbreviations

AMF: arbuscular mycorrhizal fungi
PEG: polyethylene glycol
VI: vegetative incompatibility
VCG: vegetative compatibility group
CAT: conidial anastomosis tube

## References

[1] Spatafora JW, Robbertse B, Borkovich KA, Ebbole DJ (2010). Phylogenetics and phylogenomics of the fungal tree of life. Cellular and molecular biology of filamentous fungi.

[2] Debuchy R, Berteaux-Lecellier V, Silar P, Borkovich KA, Ebbole DJ (2010). Mating systems and sexual morphogenesis in ascomycetes. Cellular and molecular biology of filamentous fungi.

[3] Weichert M, Fleißner A, van den Berg MA, Maruthachalam K (2015). Anastomosis and heterokaryon formation. Genetic transformation systems in fungi.

[4] Pontecorvo G (1956). The parasexual cycle in fungi. Annu Rev Microbiol.

[5] Read ND, Fleißner A, Roca MG, Glass NL, Borkovich KA, Ebbole DJ (2010). Hyphal fusion. Cellular and molecular biology of filamentous fungi.

[6] Roper M, Simonin A, Hickey PC, Leeder A, Glass NL (2013). Nuclear dynamics in a fungal chimera. Proc Natl Acad Sci USA.

[7] Raper JR (1966). Genetics of sexuality in higher fungi.

[8] Burgeff H (1912). Über sexualität, variabilität und vererbung bei *Phycomyces nitens*. Ber Dtsch Bot Ges..

[9] Burgeff H (1914). Untersuchungen uber variabilitat, sexualitat und erblichkeit bei *Phycomyces nitens* Kunze I. Flora.

[10] Hansen HN, Smith RE (1932). The mechanism of variation in imperfect fungi: *Botrytis cinerea*. Phytopathology.

[11] Beadle GW, Coonradt VL (1944). Heterocaryosis in *Neurospora crassa*. Genetics.

[12] Jinks JL (1952). Heterocaryosis in wild *Penicillium*. Heredity.

[13] James TY, Stenlid J, Olson Å, Johannesson H (2008). Evolutionary significance of imbalanced nuclear ratios within heterokaryons of the basidiomycete fungus *Heterobasidion parviporum*. Evolution.

[14] Rayner ADM (1991). The challenge of the individualistic mycelium. Mycologia.

[15] Shoji JY, Charlton ND, Yi M, Young CA, Craven KD (2015). Vegetative hyphal fusion and subsequent nuclear behavior in *Epichloë* grass endophytes. PLoS One.

[16] Roper M, Ellison C, Taylor JW, Glass NL (2011). Nuclear and genome dynamics in multinucleate ascomycete fungi. Curr Biol.

[17] Maheshwari R (2005). Nuclear behavior in fungal hyphae. FEMS Microbiol Lett.

[18] Marek SM, Wu J, Glass NL, Gilchrist DG, Bostock RM (2003). Nuclear DNA degradation during heterokaryon incompatibility in *Neurospora crassa*. Fungal Genet Biol.

[19] Ma L-J, van der Does HC, Borkovich KA, Coleman JJ, Daboussi M-J, Di Pietro A (2010). Comparative genomics reveals mobile pathogenicity chromosomes in *Fusarium*. Nature.

[20] Rep M, Kistler HC (2010). The genomic organization of plant pathogenicity in *Fusarium* species. Curr Opin Plant Biol.

[21] Bonfante P, Alessandro D, Lugtenberg B (2015). Arbuscular mycorrhizas: the lives of beneficial fungi and their plant hosts. Principles of plant-microbe interactions: microbes for sustainable agriculture.

[22] Jany J, Pawlowska TE (2010). Multinucleate spores contribute to evolutionary longevity of asexual Glomeromycota. Am Nat.

[23] Ropars J, Corradi N (2015). Homokaryotic vs heterokaryotic mycelium in arbuscular mycorrhizal fungi: different techniques, different results?. New Phytol.

[24] Young JPW (2015). Genome diversity in arbuscular mycorrhizal fungi. Curr Opin Plant Biol.

[25] Angelard C, Tanner CJ, Fontanillas P, Niculita-Hirzel H, Masclaux F, Sanders IR (2014). Rapid genotypic change and plasticity in arbuscular mycorrhizal fungi is caused by a host shift and enhanced by segregation. ISME J.

[26] Angelard C, Colard A, Niculita-Hirzel H, Croll D, Sanders IR (2010). Segregation in a mycorrhizal fungus alters rice growth and symbiosis-specific gene transcription. Curr Biol.

[27] Boon E, Zimmerman E, Lang BF, Hijri M (2010). Intra-isolate genome variation in arbuscular mycorrhizal fungi persists in the transcriptome. J Evol Biol.

[28] Martínez-García LB, Richardson SJ, Tylianakis JM, Peltzer DA, Dickie IA (2015). Host identity is a dominant driver of mycorrhizal fungal community composition during ecosystem development. New Phytol.

[29] Käfer E (1958). An 8-chromosome map of *Aspergillus nidulans*. Adv Genet.

[30] Pontecorvo G, Käfer E (1958). Genetic analysis based on mitotic recombination. Adv Genet.

[31] MacDonald KD, Vining LC (1983). Fungal genetics and antibiotic production. Biochemistry and genetic regulation of commercially important antibiotics.

[32] Bennett JW, Papa KE, Sidhu GS (1988). The aflatoxigenic *Aspergillus* spp. Advances in plant pathology: genetics of plant pathogenic fungi.

[33] Debets AJM, Swart K, Bos CJ (1990). Genetic analysis of *Aspergillus niger*: isolation of chlorate resistance mutants, their use in mitotic mapping and evidence for an eighth linkage group. Mol Gen Genet.

[34] Debets F, Swart K, Hoekstra RF, Bos CJ (1993). Genetic maps of eight linkage groups of *Aspergillus niger* based on mitotic mapping. Curr Genet.

[35] Ball C (1971). Haploidization analysis in *Penicillium chrysogenum*. J Gen Microbiol.

[36] Papa KE (1978). The parasexual cycle in *Aspergillus parasiticus*. Mycologia.

[37] Todd RB, Davis MA, Hynes MJ (2007). Genetic manipulation of *Aspergillus nidulans*: heterokaryons and diploids for dominance, complementation and haploidization analyses. Nat Protoc.

[38] James SW, Banta T, Barra J, Ciraku L, Coile C, Cuda Z (2014). Restraint of the G2/M transition by the SR/RRM family mRNA shuttling binding protein SNXAHRB1 in *Aspergillus nidulans*. Genetics.

[39] Zheng H, Zhang S, Zhang S, Lu L (2015). Riboflavin level manipulates the successive developmental sequences in *Aspergillus nidulans*. Curr Microbiol.

[40] Zhang J, Debets AJM, Verweij PE, Melchers WJG, Zwaan BJ, Schoustra SE (2015). Asexual sporulation facilitates adaptation: the emergence of azole resistance in *Aspergillus fumigatus*. Evolution.

[41] De la Guardia MD, Aragón CM, Murillo FJ, Cerdá-Olmedo E (1971). A carotenogenic enzyme aggregate in *Phycomyces*: evidence from quantitive complementation. Proc Natl Acad Sci USA.

[42] Sanz C, Alvarez MI, Orejas M, Velayos A, Eslava AP, Benito EP (2002). Interallelic complementation provides genetic evidence for the multimeric organization of the *Phycomyces blakesleeanus* phytoene dehydrogenase. Eur J Biochem.

[43] Leslie JF (1993). Fungal vegetative compatibility. Annu Rev Phytopathol.

[44] Aanen D, Debets A, Glass N, Saupe S, Borkovich K, Ebbole D (2010). Biology and genetics of vegetative incompatibility in fungi. Cellular and molecular biology of filamentous fungi.

[45] Micali CO, Smith ML (2003). On the independence of barrage formation and heterokaryon incompatibility in *Neurospora crassa*. Fungal Genet Biol.

[46] Puhalla JE, Hummel M (1983). Vegetative compatibility groups within *Verticillium dahliae*. Phytopathology.

[47] Ford EJ, Miller RV, Gray H, Sherwood JE (1995). Heterokaryon formation and vegetative compatibility in *Sclerotinia sclerotiorum*. Mycol Res.

[48] Puhalla JE (1985). Classification of strains of *Fusarium oxysporum* on the basis of vegetative compatibility. Can J Bot.

[49] Birkett JA, Rowlands RT (1981). Chlorate resistance and nitrate assimilation in industrial strains of *Penicillium chrysogenum*. J Gen Microbiol.

[50] Cove D, Road M (1976). Chlorate toxicity in *Aspergillus nidulans*: the selection and characterization of chlorate resistant mutants. Heredity.

[51] Brooker NL, Leslie JF, Dickman MB (1991). Nitrate non-utilizing mutants of *Colletotrichum* and their use in studies of vegetative compatibility and genetic relatedness. Phytopathology.

[52] Correll JC, Klittich CJR, Leslie JF (1987). Nitrate nonutilizing mutants of *Fusarium oxysporum* and their use in vegetative compatability tests. Phytopathology.

[53] Korolev N, Katan T (1997). Improved medium for selecting nitrate-nonutilizing (*nit*) mutants of *Verticillium dahliae*. Phytopathology.

[54] Korolev N, Katan T, Katan J (2009). Physiological races and vegetative compatibility groups among *Verticillium dahliae* isolates from tomato in Israel. Acta Hortic.

[55] Papaioannou IA, Typas MA (2015). High-throughput assessment and genetic investigation of vegetative compatibility in *Verticillium dahliae*. J Phytopathol.

[56] Aiuchi D, Inami K, Sugimoto M, Shinya R, Tani M, Kuramochi K (2008). A new method for producing hybrid strains of the entomopathogenic fungus *Verticillium lecanii* (*Lecanicillium* spp.) through protoplast fusion by using nitrate non-utilizing (*nit*) mutants. Micol Apl Int.

[57] Koike M, Shinya R, Aiuchi D, Mori M, Ogino R, El-Shemy HA (2011). Future biological control for soybean cyst nematode. Soybean physiology and biochemistry.

[58] Salman A, Shufan E, Lapidot I, Tsror L, Moreh R, Mordechai S (2015). Assignment of *Colletotrichum coccodes* isolates into vegetative compatibility groups using infrared spectroscopy: a step towards practical application. Analyst.

[59] Read ND, Goryachev AB, Lichius A (2012). The mechanistic basis of self-fusion between conidial anastomosis tubes during fungal colony initiation. Fungal Biol Rev.

[60] Fleissner A, Leeder AC, Roca MG, Read ND, Glass NL (2009). Oscillatory recruitment of signaling proteins to cell tips promotes coordinated behavior during cell fusion. Proc Natl Acad Sci USA.

[61] Leeder AC, Jonkers W, Li J, Louise Glass N (2013). Early colony establishment in *Neurospora crassa* requires a MAP kinase regulatory network. Genetics.

[62] Lichius A, Lord KM (2014). Chemoattractive mechanisms in filamentous fungi. Open Mycol J.

[63] Correll JC, Klittich CJR, Leslie JF (1989). Heterokaryon self-incompatibility in *Gibberella fujikuroi* (*Fusarium moniliforme*). Mycol Res.

[64] Anné J, Peberdy JF (1975). Conditions for induced fusion of fungal protoplasts in polyethylene glycol solutions. Arch Microbiol.

[65] Anné J, Peberdy JF (1976). Induced fusion of fungal protoplasts following treatment with polyethylene glycol. J Gen Microbiol.

[66] Minuth W, Esser K (1983). Intraspecific, interspecific, and intergeneric recombination in β-lactam producing fungi via protoplast fusion. Appl Microbiol Biotechnol.

[67] Glass NL, Dementhon K (2006). Non-self recognition and programmed cell death in filamentous fungi. Curr Opin Microbiol.

[68] Pinan-Lucarré B, Paoletti M, Clavé C (2007). Cell death by incompatibility in the fungus *Podospora*. Semin Cancer Biol.

[69] Cortesi P, Milgroom MG (1998). Genetics of vegetative incompatibility in *Cryphonectria parasitica*. Appl Environ Microbiol.

[70] Zhang DX, Spiering MJ, Dawe AL, Nuss DL (2014). Vegetative incompatibility loci with dedicated roles in allorecognition restrict mycovirus transmission in chestnut blight fungus. Genetics.

[71] Zhang DX, Spiering MJ, Choi GH, Nuss DL (2014). Genes associated with *Cryphonectria parasitica* vegetative incompatibility loci. Acta Hortic.

[72] Saupe SJ (2000). Molecular genetics of heterokaryon incompatibility in filamentous ascomycetes. Microbiol Mol Biol Rev.

[73] Glass NL, Jacobson DJ, Shiu PKT (2000). The genetics of hyphal fusion and vegetative incompatibility in fIlamentous ascomycete fungi. Biotechnology.

[74] Aanen DK, Debets AJM, de Visser JAGM, Hoekstra RF (2008). The social evolution of somatic fusion. BioEssays.

[75] Saupe SJ (2011). The [Het-s] prion of *Podospora anserina* and its role in heterokaryon incompatibility. Semin Cell Dev Biol.

[76] Paoletti M, Saupe SJ (2009). Fungal incompatibility: evolutionary origin in pathogen defense?. BioEssays.

[77] Bastiaans E, Debets AJM, Aanen DK, Van Diepeningen AD, Saupe SJ, Paoletti M (2014). Natural variation of heterokaryon incompatibility gene het-c in *Podospora anserina* reveals diversifying selection. Mol Biol Evol.

[78] Carbone I, Liu Y-C, Hillman BI, Milgroom MG (2004). Recombination and migration of *Cryphonectria hypovirus 1* as inferred from gene genealogies and the coalescent. Genetics.

[79] Gómez-Lama Cabanás C, Pérez-Artés E (2014). New evidence of intra-race diversity in *Fusarium oxysporum* f. sp. *dianthi* populations based on vegetative compatibility groups. Eur J Plant Pathol.

[80] Ghag SB, Shekhawat UKS, Ganapathi TR (2015). Fusarium wilt of banana: biology, epidemiology and management. Int J Pest Manag.

[81] Talma K, Zamir D, Sarfatti M, Katan J (1991). Vegetative compatibility groups and subgroups in *Fusarium oxysporum* f. sp. *radicis*-*lycopersici*. Am Phytopathol Soc.

[82] Grubisha LC, Cotty PJ (2010). Genetic isolation among sympatric vegetative compatibility groups of the aflatoxin-producing fungus *Aspergillus flavus*. Mol Ecol.

[83] Atehnkeng J, Donner M, Ojiambo PS, Ikotun B, Augusto J, Cotty PJ (2015). Environmental distribution and genetic diversity of vegetative compatibility groups determine biocontrol strategies to mitigate aflatoxin contamination of maize by *Aspergillus flavus*. Microb Biotechnol.

[84] Jaynes RA, Elliston JE (1980). Pathogenicity and canker control by mixtures of hypovirulent strains of *Endothia parasitica* in American Chestnut. Am Phytopathol Soc.

[85] Anagnostakis SL (1983). Conversion to curative morphology in *Endothia parasitica* and its restriction. Mycologia.

[86] Saupe SJ, Daskalov A (2012). The [Het-s] prion, an amyloid fold as a cell death activation trigger. PLoS Pathog.

[87] Seuring C, Greenwald J, Wasmer C, Wepf R, Saupe SJ, Meier BH (2012). The mechanism of toxicity in HET-S/HET-s prion incompatibility. PLoS Biol.

[88] Eichner T, Radford SE (2011). A diversity of assembly mechanisms of a generic amyloid fold. Mol Cell.

[89] Chiti F, Dobson CM (2006). Protein misfolding, functional amyloid, and human disease. Annu Rev Biochem.

[90] Bartoszewska M, Kiel JAKW (2011). The role of macroautophagy in development of filamentous fungi. Antioxid Redox Signal.

[91] Chen N, Debnath J (2010). Autophagy and tumorigenesis. FEBS Lett.

[92] Gu YH, Ko WH (2001). Creation of hybrid vigor through nuclear transplantation in *Phytophthora*. Can J Microbiol.

[93] Couteaudier Y, Viaud M, Riba G (1996). Genetic nature, stability, and improved virulence of hybrids from protoplast fusion in *Beauveria*. Microb Ecol.

[94] Davenport CB (1908). Degeneration, albinism, and inbreeding. Science.

[95] East EM. Inbreeding in corn. Reports Connect Agric. Exp. Stn. 1907. 1908. p. 419–28.

[96] Shull GH. The composition of a field of maize. Reports Am. Breeders Assoc. 1908, p. 296–301.

[97] Leitão AL, Enguita FJ (2014). Fungal extrolites as a new source for therapeutic compounds and as building blocks for applications in synthetic biology. Microbiol Res.

[98] Anné J (1982). Comparison of penicillins produced by inter-species hybrids from *Penicillium chrysogenum*. Eur J Appl Microbiol Biotechnol.

[99] Stuart W. Heterologous dimeric proteins produced in heterokaryons. 1997: U.S. Patent No. 5,643,745.

[100] Stuart W. Novel uses for Neurospora heterokaryons. In: *Neurospora* information conference, Asilomar, Califorina; 1998. p. 9 [Abstract].

[101] Hamlyn P, Ball C, Sebek OK, Laskin AI (1979). Recombination studies with *Cephalosporium acremonium*. Genetics of industrial microorganisms.

[102] Chen CW, Kan S-T, Lin L-S, Vaněk Z, Hošťálek Z (1986). Protoplast fusion studies. Overproduction of microbial metabolites.

[103] Tahoun MK (1993). Gene manipulation by protoplast fusion and penicillin production by *Penicillium chrysogenum*. Appl Biochem Biotechnol.

[104] Künkel W, Berger D, Risch S, Wittmann-Bresinsky B (1992). Genetic instability of industrial strains of *Penicillium chrysogenum*. Appl Microbiol Biotechnol.

[105] Clutterbuck JA, Arora DK (2004). Stability and instability of fungal genomes. Handbook of fungal biotechnology.

[106] Davière JM, Langin T, Daboussi MJ (2001). Potential role of transposable elements in the rapid reorganization of the *Fusarium oxysporum* genome. Fungal Genet Biol.

[107] Wang M, Liu S, Li Y, Xu R, Lu C, Shen Y (2010). Protoplast mutation and genome shuffling induce the endophytic fungus *Tubercularia* sp. TF5 to produce new compounds. Curr Microbiol.

[108] Dillon AJP, Camassola M, Henriques JAP, Fungaro MHP, Azevedo ACS, Velho TAF (2008). Generation of recombinants strains to cellulases production by protoplast fusion between *Penicillium echinulatum* and *Trichoderma harzianum*. Enzyme Microb Technol.

[109] Gandra S, Barter D, Morgan DJ, Laxminarayan R, Laxminarayan R, Teillant A (2015). Potential burden of antibiotic resistance on surgery and cancer chemotherapy antibiotic prophylaxis in the USA: a literature review and modelling study. Lancet Infect Dis.

[110] Blackwell MM (2011). The fungi: 1, 2, 3 … 5.1 million species?. Am J Bot.

[111] Yamashita F, Hotta K, Kurasawa S, Okami Y, Umezawa H (1985). New antibiotic-producing streptomycetes, selected by antibiotic resistance as a marker. I. New antibiotic production generated by protoplast fusion treatment between *Streptomyces griseus* and *S. tenjimariensis*. J Antibiot.

[112] Okamura T, Nagata S, Misono H, Nagasaki S (1989). New antibiotic-producing *Streptomyces* TT-Strain, generated by electrical fusion of protoplasts. J Ferment Bioeng.

[113] Liabsuetrakul T, Choobun T, Peeyananjarassri K, Islam Q (2007). Prophylactic use of ergot alkaloids in the third stage of labour. Cochrane Collab.

[114] Silberstein SD, Kori SH (2013). Dihydroergotamine: a review of formulation approaches for the acute treatment of migraine. CNS Drugs.

[115] Robbers J, Mizrahi A, Van Wezel AL (1984). The fermentative production of ergot alkaloids. Advances in biotechnological processes.

[116] Didek-Brumec M, Gaberc-Porekar V, Alačević M, Sočič H (1993). Strain improvement of *Claviceps purpurea* by protoplast fusion without introducing auxotrophic markers. Appl Microbiol Biotechnol.

[117] Wani MC, Taylor HL, Wall ME, Coggon P, McPhall AT (1971). Plant antitumor agents. VI. Isolation and structure of taxol, a novel antileukemic and antitumor agent from *Taxus brevifolia*. J Am Chem Soc.

[118] Zhou X, Zhu H, Liu L, Lin J, Tang K (2010). A review: recent advances and future prospects of taxol-producing endophytic fungi. Appl Microbiol Biotechnol.

[119] Zhao K, Sun L, Ma X, Li X, Wang X, Ping W (2013). Improved taxol production in *Nodulisporium sylviforme* derived from inactivated protoplast fusion. Afr J Biotechnol.

[120] Zhao K, Ping W, Zhang L, Liu J, Lin Y, Jin T (2008). Screening and breeding of high taxol producing fungi by genome shuffling. Sci China Ser C Life Sci.

[121] Ushijima S, Nakadai T (1987). Breeding by protoplast fusion of koji mold, *Aspergillus sojae*. Agric Biol Chem.

[122] Ushijima S, Nakadai T, Uchida K (1990). Breeding of new koji-molds through interspecific hybridization between *Aspergillus oryzae* and *Aspergillus sojae* by protoplast fusion. Agric Biol Chem.

[123] Ushijima S, Nakadai T, Uchida K (1991). Interspecific electrofusion of protoplasts between *Aspergillus oryzae* and *Aspergillus sojae*. Agric Biol Chem.

[124] Xu D, Pan L, Zhao H, Zhao M, Sun J, Liu D (2011). Breeding and identification of novel koji molds with high activity of acid protease by genome recombination between *Aspergillus oryzae* and *Aspergillus niger*. J Ind Microbiol Biotechnol.

[125] Kida K, Kume K, Morimura S, Sonoda Y (1992). Repeated-batch fermentation process using a thermotolerant flocculating yeast constructed by protoplast fusion. J Ferment Bioeng.

[126] Takaki M, Tan L, Murakami T, Tang Y-Q, Sun Z-Y, Morimura S (2015). Production of biofuels from sweet sorghum juice via ethanol–methane two-stage fermentation. Ind Crops Prod.

[127] Varavallo MA, de Queiroz MV, Lana TG, de Brito ATR, Gonçalves DB, de Araújo EF (2007). Isolation of recombinant strains with enhanced pectinase production by protoplast fusion between *Penicillium expansum* and *Penicillium griseoroseum*. Braz J Microbiol.

[128] Solís S, Loeza J, Segura G, Tello J, Reyes N, Lappe P (2009). Hydrolysis of orange peel by a pectin lyase-overproducing hybrid obtained by protoplast fusion between mutant pectinolytic *Aspergillus flavipes* and *Aspergillus niveus* CH-Y-1043. Enzyme Microb Technol.

[129] Hassan MM (2014). Influence of protoplast fusion between two *Trichoderma* spp. on extracellular enzymes production and antagonistic activity. Biotechnol Biotechnol Equip.

[130] Kim JS, Skinner M, Gouli S, Parker BL (2011). Generating thermotolerant colonies by pairing *Beauveria bassiana* isolates. FEMS Microbiol Lett.

[131] Foster RC (1988). Microenvironments of soil microorganisms. Biol Fertil Soils.

[132] Harms H, Schlosser D, Wick LY (2011). Untapped potential: exploiting fungi in bioremediation of hazardous chemicals. Nat Rev Microbiol.

[133] Mitra J, Mukherjee PK, Kale SP, Murthy NBK (2001). Bioremediation of DDT in soil by genetically improved strains of soil fungus *Fusarium solani*. Biodegradation.

[134] Patil NS, Patil SM, Govindwar SP, Jadhav JP (2015). Molecular characterization of intergeneric hybrid between *Aspergillus oryzae* and *Trichoderma harzianum* by protoplast fusion. J Appl Microbiol.

[135] Lazova R, LaBerge GS, Duvall E, Spoelstra N, Klump V, Sznol M (2013). A melanoma brain metastasis with a donor-patient hybrid genome following bone marrow transplantation: first evidence for fusion in human cancer. PLoS One.

[136] Berndt B, Zanker KS, Dittmar T (2013). Cell fusion is a potent inducer of aneuploidy and drug resistance in tumor cell/normal cell hybrids. Crit Rev Oncog.

[137] Ghabrial SA, Castón JR, Jiang D, Nibert ML, Suzuki N (2015). 50-Plus years of fungal viruses. Virology.

[138] Dana GV, Cooper AM, Pennington KM, Sharpe LM (2014). Methodologies and special considerations for environmental risk analysis of genetically modified aquatic biocontrol organisms. Biol Invasions.

[139] Rodriguez-Iglesias A, Schmoll M, van den Berg MA, Maruthachalam K (2004). Protoplast transformation for genome manipulation in fungi. Genetic transformation systems in fungi.

[140] Kale SP, Bhatnagar D, Arora DK (2004). Protoplast isolation, regeneration, and fusion in filamentous fungi. Handbook of fungal biotechnology.

